# Assessing knowledge, acts of discrimination, stigmatizing attitudes and its associated factors towards people living with HIV (PLHIV) among Family Medicine trainees in Malaysia

**DOI:** 10.51866/oa1298

**Published:** 2022-07-20

**Authors:** Hiang Ngee Chan, Anuar Mohamad, Aneesa Abdul Rashid, Bee Kiau Ho, Alia Abdul Aziz Cooper, Haslina Mukhtar Aajamer, Ermi Noor Emjah, Jashithra Syamala Krishnan, Gloria Neo Lih Hwee

**Affiliations:** 1 MBBS (IMU), FRACGP (Australia) Klinik Kesihatan Cheras, Jalan Yaacob Latif, Bandar Tun Razak, Kuala Lumpur, Malaysia. Email: hiangngee@gmail.com; 2 MD (UKM), Dr. Fam. Med (UKM) Klinik Kesihatan Cheras, Jalan Yaacob Latif, Bandar Tun Razak, Kuala Lumpu, Malaysia.; 3 MBBCh BAO (NUI), DrFamMed (UKM) Department of Family Medicine, Faculty of Medicine & Health Sciences, Universiti Putra Malaysia (UPM), UPM, Serdang, Malaysia.; 4 MBBS (UM), MMed (Family Medicine) Klinik Kesihatan Bandar Botanic, Jalan Langat, Bandar Botanic, Klang, Selangor, Malaysia.; 5 MBChB (University of Dundee), FRACGP (Australia) Klinik Kesihatan Kota Damansara 40-70, Jalan Pekaka 8/3, Kota Damansara, Petaling Jaya, Selangor, Malaysia.; 6 MD (USU), Graduate Certificate of Family Medicine (AFPM) Klinik Medina, Subang Jaya, Malaysia.; 7 MD (USM), Diploma in Family Medicine (AFPM) Pejabat Kesihatan Daerah Hulu Langat, Kajang, Selangor, Malaysia.; 8 MBBS (AIMST), Diploma in Family Medicine (AFPM) Klinik Kesihatan Pandamaran, Persiaran Raja Muda Musa, Klang, Selangor, Malaysia.; 9 MD (CSMU), Graduate Certificate in Family Medicine (AFPM) Klinik Alam Medic, Taman Sri Muda, Subang Jaya, Selangor, Malaysia.

**Keywords:** Stigmatisation, Discrimination, HIV patients, Primary care doctors

## Abstract

**Introduction:**

Human immunodeficiency virus (HlV)-related stigmatisation and discrimination adversely affect health outcomes in terms of timely diagnosis, treatment and care. Despite global efforts, they remain common among healthcare workers worldwide. In Malaysia, family medicine specialists are entrusted with the care of HIV patients at the primary care level. This study aimed to assess HIV-related knowledge, acts of discrimination, stigmatising attitudes and their associated factors among family medicine trainees in Malaysia.

**Methodology:**

This cross-sectional study was conducted among 397 family medicine trainees in Malaysia using a validated, self-administered questionnaire that assessed the participants’ sociodemographic information, HIV/AIDS knowledge, stigmatising attitudes (attitudes of blame, attitudes towards imposed measures, comfort in dealing with HIV patients) and acts of discrimination.

**Results:**

The most common stigmatisation was “attitudes of blame” (mean [SD] score: 3.0 (0.74); range score:1–5), and the most frequent discriminatory act was breaching patient confidentiality (54.9%). Around 82.1% had good knowledge of HIV/AIDS. Married participants and participants who had 7 years or less in service were more stigmatising in “attitudes of imposed measures” towards people living with HIV (p=0.006).

**Conclusion:**

Family medicine trainees exhibited stigmatisation and discrimination towards HIV patients despite having good HIV knowledge. Hence, appropriate and concerted health education should be given to all family medicine trainees to eliminate stigmatisation and discrimination.

## Introduction

HIV/AIDS is a major health burden worldwide. Globally, an estimated 38 million people are living with this virus, of whom 5.8 million are living in the Asia Pacific region.^1^ Malaysia has 87,041 people living with HIV (PLHIV).^2^ Malaysia has one of the lowest HIV prevalences in the Asia Pacific region, at 0.4% in a population of 32.385 million.

Despite being a global pandemic for over 30 years, HIV/AIDS-related stigmatisation still exists among health care workers (HCWs) in many regions worldwide.^3-5^ Stigmatisation is defined as negative attitudes and beliefs about PLHIV, and discrimination is behaviour that results from these beliefs.^5^ Surveys conducted by The People Living with HIV (PLHIV) Stigma Index among PLHIV in 19 countries between 2011 and 2016 revealed that denial of healthcare service, provision of substandard care, coerced procedures (including HIV testing), imposed conditions in exchange for treatment, and breach of confidentiality remained common judgmental acts committed towards HIV patients.^4^ A survey conducted in Malaysia in 2012 showed the presence of similar HIV-related stigmatisation and discrimination among HCWs.^6^ Common judgmental acts among HCWs in Belize included attitudes of blame and breaching of confidentiality.^7^ A study in Thailand found HCWs agreeing with coerced sterilisation among HIV-positive women, unwillingness to provide care and giving substandard care to PLHIV.^8^

HIV-related stigma and discrimination are known to adversely affect health behaviour in PLHIV and exist as challenging barriers to timely HIV testing, treatment and care. A meta-analysis of 64 studies in 2016 found significant associations between HIV-related stigma and higher rates of depression, lower social support, lower medical adherence, and less usage of health and social services.^9^ A few systematic reviews have confirmed that HIV-related stigma was associated with late presentation for HIV care and compromised medical adherence.^10,11^ Late testing, delayed initiation of treatment and medication non-adherence inevitably impede viral load suppression among HIV patients, leading to poor health outcomes and hampering efforts in reducing HIV transmission.

Given that stigmatisation and discrimination stand as barriers to HIV testing and treatment, studying their associated factors is fundamental. These judgemental attitudes were often due to factors such as the association of HIV infection with perceived moral and religious misconduct (sexual promiscuity, homosexuality, drug use, etc.), poor HIV knowledge, fear of transmission and lack of experience in treating HIV patients.^12,13,14,15^ Andrewin et al. (2008) reported that having formal HIV/ AIDS training and being aware of HIV policy were significantly associated with less stigmatisation in Belize.^7^ HCWs in Asia, including Malaysia, and Africa have been reported to have good knowledge of HIV, but they may need improved knowledge on its modes of transmission.^16,17,18,19^

A local study by Chew et al. (2013) revealed that HIV-related stigmatisation among medical students was associated with less clinical exposure to HIV patients and poor HIV knowledge.^20^ Tee et al. (2019) revealed the presence of discriminatory intent among physicians in Malaysia, which was associated with more negative feelings towards PLHIV.^21^ In Malaysia, family physicians are entrusted with the provision of care for PLHIV at the primary care level. Interestingly, to date, no study has assessed the knowledge, stigmatisation and discriminatory acts towards HIV/AIDS patients among family physicians in Malaysia.

Hence, this study aimed to determine the knowledge and awareness of HIV, acts of discrimination, stigmatising attitudes towards PLHIV and their associated factors (including sociodemographic factors, clinical experience, training in HIV/AIDS and primary care, and religiosity) among family medicine trainees in Malaysia. Information obtained from this study could provide a baseline understanding of HIV-related stigmatisation and discrimination among family medicine trainees. Such invaluable information could help in designing concerted health education targeting to reduce stigmatisation and discrimination, thereby improving HIV preventive care, timely diagnosis and treatment.

## Methods

### Study design

This was a cross-sectional study conducted among Advanced Training of Family Medicine (ATFM) trainees and Graduate Certificate in Family Medicine (GCFM) trainees who attended the GCFM and ATFM workshops in March 2019 and May 2019, respectively. Both programmes are part of the parallel pathway to the family medicine specialisation in Malaysia. GCFM is the initial 2-year training of the parallel pathway, followed by the further 2-year ATFM training. Completion of ATFM is a prerequisite for family medicine trainees to sit for the conjoint Membership of the Academy of Family Physicians of Malaysia (MAFP) and Fellowship of Royal Australian College of General Practitioners (FRACGP) examinations. The award of the conjoint examination is equivalent to the family medicine specialist qualification in Malaysia.

### Sample size

This study used universal sampling. A total of 454 family medicine trainees who attended the GCFM and ATFM workshops on 23^rd^ March 2019 and 25^th^ May 2019, respectively, were invited to participate.

### Study instrument

This study used a self-administered questionnaire that consisted of four sections (I—IV). Section I assessed the participant’s sociodemographic details such as age, gender, religiosity, marital status, years of working in the medical profession, ATFM/GCFM trainee intake, whether the participant provided care to HIV/AIDS patients, whether the participant had any formal HIV/ AIDS training, the number of HIV/AIDS cases encountered in the past 6 months and awareness of HIV test policy.

Section II assessed the participants’ knowledge of HIV/AIDS through six items with ‘true’, ‘false’ or ‘don’t know’ answers. A score of 1 was given for a correct answer and 0 for incorrect or ‘don’t know’ answers. The total score ranged between 0 and 6. A score of 4 or above was considered good knowledge, whereas a score of 3 or below was considered poor knowledge.

Section III evaluated stigmatising attitudes towards HIV/AIDS patients, comprising three domains: (1) attitudes towards imposed measures (four items); (2) attitudes of blame/judgement (three items); and (3) Comfortableness in dealing with HIV/AIDS patients (two items). These items had 5-point Likert scale responses, ranging from strongly disagree (score of 1) to strongly agree (score of 5). Reverse coding was applied to both items under “comfortableness in dealing with HIV/ AIDS patients”. A score of 1 was regarded as least stigmatising while 5 was considered most stigmatising. These items were summed and averaged to obtain the stigmatising scores for each subscale.

Section IV evaluated the acts of discrimination through five questions. The responses were recorded according to a 5-point Likert-type scale, which ranged from 1 (never) to 5 (all of the time). A score of 1 was regarded as least discriminatory and 5 as most discriminatory. Reverse coding was applied to items 1 and 5. A higher total score indicated a higher frequency of discriminatory acts.

This questionnaire was validated for use among doctors and nurses in Belize by Andrewin and Chien.^7^ Reliability testing was done to determine the consistency. For section III, Cronbach’s a for the three domains:(1) Attitudes toward imposed measures, (2) Attitudes of blame / judgment and (3) Comfortableness in dealing with HIV/AIDS patients were 0.71, 0.60 and 0.83, respectively. However, the answer for one of the questions under section II was modified from “true” to “false” based on expert panel input and literature review. The question was: “After needle stick injury with a needle from an HIV-infected patient, immediately gently expressing blood from the puncture site reduces the risk of contracting HIV infection.” Permission to use this questionnaire was obtained.

### Ethical approval

Approval to conduct the study was obtained from the Medical Research and Ethics Committee (MREC) of the Ministry of Health Malaysia (NMRR-18-3351-44916). The participants were required to sign an informed consent before participating in the study. Their anonymity was maintained throughout the research process.

### Data analysis

Data were analysed using IBM SPSS version 25. In the descriptive analysis, the data were presented as mean and standard deviation (SD). Categorical variables were described as frequency (n) and percentage (%). The associations between independent variables and stigmatising attitudes towards PLHIV were examined using simple logistic regression. The independent variables with a p-value of <0.25 from the bivariate analysis were selected for multiple logistic regression analysis to determine their independent association with the dependent variables. The significance level was set at p<0.05.

## Results

Out of 454 attendees of the ATFM and GCFM workshops, 417 family medicine trainees consented and participated in this study. The response rate for this study was 91.9%. However, 20 participants were excluded from the analysis because of incomplete questionnaires. Therefore, only data obtained from 397 participants were analysed.

**Table 1:** Sociodemographic characteristics of the participants (n=397)

Variables	n (%)	Mean (SD)
*Age (years), mean (SD)*		33.4 (4.0)
*Gender*		
Male	100 (25.2)	
Female	297 (74.8)	
*Nationality*		
Malaysian	395 (99.5)	
Other	2 (0.5)	
*Marital status*		
Single	121 (30.5)	
Married	276 (69.5)	
*Clinical experience*		
*Number of years in service, mean (SD)*		7.4 (3.17)
*Need to manage HIV patients*		
Yes	231 (58.2)	
No	166 (41.8)	
*Number of HIV cases encountered in the past 6 months*		
≤10	384 (96.7)	
>10	13 (3.3)	
*Awareness of HIV testing policy*		
Yes	299 (75.3)	
No/don’t know	98 (24.7)	
*Training*		
*Formal HIV/AIDS training*		
Yes	106 (26.7)	
No	291 (73.3)	
*Family medicine/primary care training*		
ATFM programme	70 (17.6)	
GCFM programme^a^	327 (82.4)	
*Religiosity*		
Somewhat/very	369 (92.9)	
No	28 (7.1)	

* All continuous data were normally distributed.

a ATFM = Advanced Training in Family Medicine programme. GCFM = Graduate Certificate in Family Medicine programme.

### Participant characteristics

Table 1 shows the sociodemographic information, clinical experience, training and religiosity of the participants.

*Sociodemographic data*

The mean (SD) age of the participants was 33.4 (4.0) years. More than two thirds of the respondents were female (74.8%) and married (69.5%).

*Clinical experience*

More than half (58.2%) of participants were directly involved in managing HIV/AIDS patients. Most (96%) had encountered 10 or fewer HIV cases in the past 6 months, and around 75.3% were aware of the HIV testing policy in Malaysia.

*Training in HIV/AIDS and primary care*

Approximately one quarter of the participants had received formal training in managing HIV/ AIDS patients. Most were GCFM programme trainees.

*Religiosity*

The majority of participants indicated being religious, and only 7.1% denied being religious.

**Figure 1:**
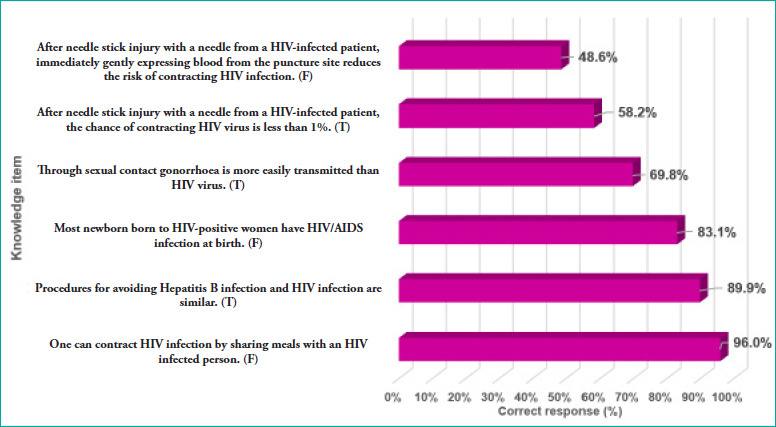
Family physician trainees’ knowledge of HIV

### Knowledge ofHIV/AIDS

**Figure 1** shows that the mean score (SD) for knowledge of HIV/AIDS was 4.46 (1.08), which was above the midpoint of 3.0 from the total score range (0-6). Around 82.1% of the participants had good knowledge of HIV/AIDS. The majority answered the following three items correctly: “Most newborns born to HIV-positive women have HIV/AIDS infection at birth”, “Procedures for avoiding Hepatitis B and HIV infection are similar” and “One can contract HIV infection by sharing meals with an HIV-infected person”.

**Table 2:** Stigmatising attitudes and acts of discrimination towards HIV/ AIDS patients among family physician trainees

Stigmatising attitudes subscales and items	No. of respondents (%)					
	Strongly disagree^a^	Disagree	No opinion	Agree	Strongly agree	Mean (SD)
*Attitudes of blame/judgement*						**3.00 (0.74)**
I feel more sympathetic towards people who get AIDS from blood transfusions than those who get it from intravenous drug abuse.	16 (4.0)	57 (14.4)	66 (16.6)	161 (40.6)	97 (24.4)	3.67 (1.11)
I feel that if a child contracts the HIV/AIDS virus from their mother through mother-to-child or vertical transmission, the mother is to be blamed for the child’s disease.	54 (13.6)	187 (47.1)	88 (22.2)	49 (12.3)	19 (4.8)	2.48 (1.03)
I have little sympathy for people who get AIDS from sexual promiscuity.	33 (8.3)	131 (33.0)	115 (29.0)	97 (24.4)	21 (5.3)	2.85 (1.05)
*Attitudes toward imposed measures*						**2.57 (0.74)**
All patients admitted to the hospital should be HIV-tested.	50 (12.6)	211 (53.1)	34 (8.6)	80 (20.2)	22 (5.5)	2.53 (1.11)
Relatives/sexual partners of patients with HIV/ AIDS should be notified of the patient’s status even without his/her consent.	78 (19.7)	149 (37.5)	24 (6.0)	91 (22.9)	55 (13.9)	2.74 (1.37)
Patients with HIV/AIDS should be cared for and treated in their own hospitals and facilities, away from other patients who do not have HIV/AIDS.	92 (23.2)	239 (60.2)	22 (5.5)	39 (9.8)	5 (1.3)	2.06 (0.89)
A health professional with HIV/AIDS should not be working in any area of health care that requires patient contact.	51 (12.8)	134 (33.8)	39 (9.8)	123 (31.0)	50 (12.6)	2.97 (1.29)
*Comfortableness dealing with HIV/AIDS patients*						**2.10 (0.75)**
I am comfortable providing health services to clients who are HIV-positive.^b^	2 (0.5)	20 (5.0)	29 (7.3)	242 (61.0)	104 (26.2)	1.93 (0.76)
I am comfortable putting a drip in someone who is showing signs of AIDS.^b^	6 (1.5)	49 (12.3)	55 (13.9)	224 (56.4)	63 (15.9)	2.27 (0.93)
*Acts of discrimination items*	Never^a^	A little of tde time	Some of tde time	Most of tde time	All tde time	Mean (SD)
I give the same amount of attention to all my patients regardless of their HIV status.^b^	1 (0.3)	6 (1.5)	34 (8.6)	142 (35.8)	214 (53.9)	1.58 (0.73)
Do you ever disclose a patient’s HIV status to a colleague who is not directly involved in the management of that case?	179 (45.1)	102 (25.7)	89 (22.4)	21 (5.3)	6 (1.5)	1.92 (1.01)
Because I suspect a patient to be HIV-positive, I let another health care worker deal with that patient.	348 (87.7)	31 (7.8)	14 (3.5)	3 (0.8)	1 (0.3)	1.18 (0.54)
Do you ever disclose a patient’s HIV status to a friend?	324 (81.6)	46 (11.6)	23 (5.8)	3 (0.8)	1 (0.3)	1.26 (0.62)
I get consent from the patient before testing his/her blood for HIV.^b^	6 (1.5)	4 (1.0)	9 (2.3)	66 (16.6)	312 (78.6)	1.30 (0.71)

a The score for responses was: “Strongly disagree” = 1, “Disagree” = 2, “No opinion” = 3, “Agree” = 4, “Strongly agree” = 5 “Never” = 1, “A little of the time” = 2, “Some of the time” = 3, “Most of the time” = 4, “All the time” = 5

b Reverse coding was applied to these questions. SD = standard deviation

### Stigmatising attitudes and acts of discrimination

Table 2 shows the subscale “attitudes of blame/judgement” had the highest stigmatising score (mean [SD] score: 3.00 [0.74]), while the least stigmatising subscale was “comfortableness in dealing with HIV/AIDS patients” (mean [SD] score: 2.10 [0.75]). Item 1 under the subscale “attitudes of blame/judgement” had the highest stigmatising score: 65% of the participants felt more sympathetic towards people who had contracted AIDS from blood transfusions than those who had contracted it from intravenous drug use (mean [SD] score: 3.67 [1.11]).

The commonest committed act of discrimination was disclosing an HIV patient’s status to a colleague not directly involved in the management of that case (Item 2); 54.9% of participants admitted that they had breached a patient’s confidentiality. Only 12.3% of participants let another HCW deal with a patient who was suspected to have HIV (**Table 2**).

**Table 3:** Simple logistic regression — factors associated with stigmatising attitudes towards HIV/ AIDS patients among family medicine trainees (stigmatising=1, non-stigmatising=0)

Variables	Attitudes towards imposed measures			Attitudes of blame/judgement			Comfortableness in dealing with HIV/AIDS patients		
	OR	95% Cl	p-value	OR	95% Cl	p-value	OR	95% Cl	p-value
**Gender**									
Male	1.16	0.61-2.20	0.662	0.83	0.52-1.31	0.414	0.61	0.36-1.04	0.068
Female	1.00			1.00			1.00		
**Nationality**									
Malaysian	5.58	0.35-90.48	0.226	1.53	0.10-24.67	0.764	671667388	0.00-0.00	0.999
Other	1.00			1.00			1.00		
**Religiosity**									
Somewhar/very	0.64	0.19-2.20	0.482	0.10	0.45-2.17	0.977	0.86	0.38-1.97	0.724
No	1.00			1.00			1.00		
**Marital status**									
Single	**0.53**	**0.30-0.93**	**0.027**	0.82	0.53-1.26	0.355	0.73	0.45-1.18	0.200
Married	**1.00**			1.00			1.00		
**Batch**									
ATFM	0.75	0.38-1.48	0.414	0.79	0.47-1.33	0.372	1.33	0.77-2.31	0.305
GCFM	1.00			1.00			1.00		
**Need to manage HIV patients**									
Yes	0.82	0.47-1.43	0.480	0.93	0.62-1.40	0.732	1.26	0.81-1.96	0.314
No	1.00			1.00			1.00		
**Formal HIV/AIDS training**									
Yes	1.59	0.81 3.11	0.180	1.24	0.78-1.96	0.364	0.83	0.50-1.36	0.459
No	1.00			1.00			1.00		
**Awareness of HIV testing policy**									
Yes	0.63	0.31-1.26	0.193	0.96	0.60-1.53	0.857	0.67	0.41-1.09	0.104
No/don’t know	1.00			1.00			1.00		
**Age**									
≤33 years	1.16	0.67-2.01	0.597	0.80	0.55-1.20	0.278	1.087	0.70-1.69	0.709
>33 years	1.00			1.00			1.00		
**Number of years in service**									
≤7 years	**1.77**	**1.02-3.08**	**0.042**	1.08	0.72-1.62	0.708	0.98	0.64 1.51	0.928
>7 years	**1.00**			1.00			1.00		
**Number of HIV cases (past 6 months)**									
≤10	0.45	0.06-3.53	0.447	1.82	0.6-5.52	0.290	2.32	0.51 10.64	0.278
>10	1.00			1.00			1.00		
**HIV knowledge**									
≥67	0.88	0.43-1.84	0.741	0.64	0.37-1.1	0.105	1.16	0.65-2.06	0.615
<67	1.00			1.00			1.00		

* Significance: p<0.05

**Table 4:** Multiple logistic regression — factors associated with stigmatising attitudes towards HIV/AIDS patients among family medicine trainees (stigmatising=1, non-stigmatising=0)

	Attitudes towards imposed measures			Comfortableness in dealing with HIV/AIDS patients		
	OR	95% CI	p-value	OR	95% CI	p-value
**Marital status**						
Single	**0.44**	**0.24-0.79**	**0.006**	0.77	0.47-1.25	0.288
Married						
**Gender**						
Male				0.61	0.35-1.04	0.070
Female						
**Formal HIV/AIDS training**						
Yes	1.91	0.95-3.84	0.069			
No						
**Awareness of HIV testing policy**						
Yes	0.51	0.25-1.06	0.070	0.64	0.40-1.05	0.076
No/don’t know						
**Number of years in service**						
≤7 years	**2.73**	**1.27-4.08**	**0.006**			
>7 years						

* Significance: p<0.05

### Factors associated with HIV/AIDS stigmatising attitudes

Bivariate analysis showed that marital status and number of years in service were significantly associated with attitudes towards imposed measures. Single participants were less stigmatising towards PLHIV in terms of attitudes towards imposed measures (OR 0.53; 95% CI 0.30-0.93, p=0.027) compared to married participants. Participants who had 7 years or less in service had more stigmatising attitudes towards imposed measures (OR 1.77; 95% CI 1.02-3.08, p=0.042) compared to those with more than 7 years in service (**Table 3**).

In multivariate analysis, both marital status and number of years in service remained significantly associated with attitudes towards imposed measures (p=0.006; **Table 4**).

## Discussion

### HIV knowledge among family medicine trainees

In this study, 82.1% of family physician trainees had good HIV-related knowledge. This was likely attributable to more clinical exposure to HIV patients among our participants.^20^ However, a study by Andrewin et al. (2008) in Belize showed that 57% of 230 doctors and nurses had good HIV knowledge.^7^ It also showed that 97% of their participants correctly answered the question on casual contact, which was similar to our study, where 96% answered correctly. Another study performed in Vientiane, Laos, by Vorasane et al. (2017) found that only 56% of doctors and 60.5% of nurses had good HIV knowledge.^15^ However, a direct comparison could not be made with this study because a different tool was used to assess participants’ knowledge.

### HIV-related stigmatisation among family medicine trainees

This study revealed the presence of stigmatisation of HIV patients among family medicine trainees in Malaysia, of which “attitudes of blame” was the commonest. Participants’ moral beliefs and negative feelings towards PLHIV could have caused these judgemental thoughts.^14^ It has been well-established that HCWs are more stigmatising towards HIV patients from marginalised groups such as intravenous drug users, commercial sex workers, and homosexuals.^11,12^ This finding is consistent with a study done in Belize.^7^ As such, it is fundamental to address issues pertaining to stereotyping HIV patients among HCWs.

As for “attitudes towards imposed measures”, 43.6% of the participants agreed that a health professional with HIV/AIDS should not be working in any department that required patient contact. Possible reasons are HIV transmission—related fears and misconceptions.^22^ This finding is consistent with a study done by Reis et al. (2005) in Nigeria, where 40% of the HCWs had a similar belief.^23^ Our results also revealed HIV testing and confidentiality issues: 25.7% agreed with mandatory HIV testing for all hospitalised patients, and 36.8% agreed that relatives and sexual partners of patients should be notified even without the patient’s consent. These coercive measures are driven by HIV transmission misconceptions, attitudes of blame and symbolic stigma.^14^ Likewise, in Belize, 50.1% of HCWs agreed with mandatory HIV testing on admission to hospital, and 44.4% agreed with disclosing HIV patients’ status to relatives or sexual partners without the patient’s consent.^7^ Another study done by Gledovic et al. (2015) in Montenegro showed that 64.7% of HCWs agreed with mandatory HIV testing for all hospitalised patients, and 90% believed that all HIV patients were obliged to disclose their status to an HCW.^24^ A lack of knowledge was believed to have reinforced fear, causing these stigmatising behaviours.^24^

Over 80% of the participants were comfortable dealing with HIV/AIDS patients, consistent with the study done by Andrewin et al. (2008) in Belize.^7^ Such confidence in managing HIV patients was usually attributed to related to clinical experience.^14^

### Acts of discrimination towards HIV patients among family medicine trainees

The majority of participants discriminated against HIV patients infrequently, consistent with the study done in Belize.^7^ This was due to participants’ experience in managing HIV patients, good HIV knowledge and being more advanced in training.^14,15,25^ However, a study in India showed that the majority of the HCWs had discriminatory intent when providing care in clinical situations with fluid exposure. This was largely caused by transmission-related fears and misconceptions, attitudes of blame and negative feelings towards PLHIV.^14,24^ Similarly, a recent Malaysian study revealed that HCWs in hospitals and primary care clinics had neutral to negative attitudes and fair to poor practices towards PLHIV, possibly attributed to fear of contracting HIV.^19^

### Factors associated with HIV/AIDS stigmatising attitudes

This study demonstrated that married participants were more stigmatising in attitudes towards imposed measures towards HIV/AIDS patients, which is in keeping with the findings of a study done by Harapan et al. (2013) in Indonesia.^25^ It was postulated that married HCWs were more attentive towards their own families, resulting in more judgemental attitudes.^25^ Our study also revealed that HCWs with more than 7 years in service were less stigmatising in attitudes towards imposed measures, consistent with the findings of Dong et al.^26^ This was because HCWs developed more experience and familiarity with HIV/AIDS through more clinical encounters over time, thereby becoming more willing to provide better care, as found in studies done in Malaysia, India and Vientiane.^14,15,16^

This study has a few limitations. Firstly, it was a cross-sectional study, so causal relationships could not be established. Secondly, social desirability bias and recall bias were possible because a self-administered questionnaire was used, which might influence the accuracy of the findings. Finally, our study only recruited family medicine trainees from the parallel pathway for this specialisation, excluding trainees enrolled in the government-based master’s programme and other categories of HCWs. This could limit the generalisation of the study findings.

Nonetheless, this was the first study done in Malaysia to determine knowledge and assess acts of discrimination and stigmatising attitudes and their associated factors among family medicine trainees. Thus, this study provided a baseline measurement of HIV knowledge, stigmatisation and discrimination towards HIV patients among these trainees in Malaysia for future studies. Furthermore, as family medicine trainees, the participants will provide care to HIV patients at the primary care level in the future, and the results of this study may be used as guidance for further educational interventions for them. Finally, this study used a validated questionnaire to ensure reliability.

## Conclusion

Family medicine trainees exhibited stigmatisation and discrimination towards HIV patients. “Attitudes of blame” was the most common stigmatising behaviour, whereas acts of discrimination happened infrequently. Breaching confidentiality was the most common discriminatory act. The majority of the participants had good HIV knowledge. Higher levels of stigmatising attitudes were associated with being married and having fewer years in service. Hence, appropriate and concerted health education should be given to all family medicine trainees to eliminate stigmatisation and discrimination. Future studies should assess HIV-related judgemental behaviour among family medicine trainees from the master’s programme and other categories of HCWs to obtain a more comprehensive understanding of HIV-related stigmatisation and discrimination.
